# Buprenorphine Treatment for Opioid Use Disorder in Non–Addiction Specialty Settings

**DOI:** 10.1001/jamanetworkopen.2025.43543

**Published:** 2025-11-13

**Authors:** Sophia Huebler, Audrey L. Jones, Hongwei Zhao, Richard E. Nelson, Hildi J. Hagedorn, Eric J. Hawkins, Adam J. Gordon

**Affiliations:** 1 Program for Addiction Research, Clinical Care, Knowledge, and Advocacy, Division of Epidemiology, Department of Internal Medicine, University of Utah School of Medicine, Salt Lake City; 2 Veterans Administration Health Systems Research Informatics, Decision-Enhancement, and Analytic Sciences Center, VA Salt Lake City Health Care System, Salt Lake City, Utah; 3 Intermountain Healthcare Department of Population Health Sciences, Spencer Fox Eccles School of Medicine, University of Utah, Salt Lake City; 4 Division of Occupational and Environmental Health, Spencer Fox Eccles School of Medicine, University of Utah, Salt Lake City; 5 VA Center for Care Delivery and Outcomes Research, Minneapolis VA Health Care System, Minneapolis, Minnesota; 6 Department of Psychiatry and Behavioral Sciences, University of Minnesota, Minneapolis; 7 Center of Excellence in Substance Addiction Treatment and Education, VA Puget Sound Health Care System, Seattle, Washington; 8 Health Services Research Seattle Center of Innovation for Veteran-Centered and Value-Driven Care, Veterans Affairs Puget Sound Health Care System, Seattle, Washington; 9 Department of Psychiatry and Behavioral Sciences, University of Washington, Seattle

## Abstract

This cohort study evaluates changes in prescribing of buprenorphine treatment for opioid use disorder at primary care, mental health, and pain clinics and substance use disorder clinics in Stepped Care for Opioid Use Disorder Train the Trainer (SCOUTT) and non-SCOUTT facilities.

## Introduction

Increasing patient access to buprenorphine medication for opioid use disorder (B-MOUD) is a national priority for sustaining progress in preventing deaths from opioid use disorder (OUD). The historical relegation of OUD care to substance use disorder (SUD) addiction specialty care clinics has created access barriers. Experts advocate treating OUD in commonly used health care touchpoints, particularly office-based primary care, mental health, and pain (PC-MH-P) clinics, where patients receive longitudinal health services that may overcome SUD addiction specialty care barriers.^1^

In 2018, the Department of Veterans Affairs (VA) established the Stepped Care for Opioid Use Disorder Train the Trainer (SCOUTT) Initiative with a goal to increase B-MOUD prescribing in PC-MH-P settings.^2^ Clinical teams from 18 multifacility health care systems initially participated in SCOUTT voluntarily or by direction of the VA central office. They received in-person training on buprenorphine prescribing practices, and in 2020, another 19 facilities were added through a series of conferences. We have previously demonstrated that SCOUTT interventions (eg, external implementation facilitation, education, mentoring) improve B-MOUD prescribing among specific PC-MH-P clinics in SCOUTT-intervened facilities compared with similar PC-MH-P clinics in nonintervened settings.^3,4^ SCOUTT coevolved amid policy changes aimed at B-MOUD access expansion, notably the 2023 rescindment of the X-waiver certification process to prescribe B-MOUD. We describe compositional changes in the proportions of B-MOUD prescribing from PC-MH-P and SUD clinics in SCOUTT and non-SCOUTT facilities.

## Methods

This retrospective cohort study of B-MOUD prescribing in VA facilities from January 2016 through December 2024 used electronic health records from the Corporate Data Warehouse. We followed the STROBE reporting guideline. The VA Salt Lake City institutional review board waived the requirement to approve the project as it was a quality improvement activity and used deidentified data.

Included patients had at least 1 OUD *ICD-10* code (eTable 1 in Supplement 1) and received an outpatient B-MOUD prescription between 2016 and 2024. We evaluated all available B-MOUD prescribed to the cohort at the prescription level and identified settings (eg, SUD, PC, MH, or P) using 3-digit location identifiers (eTable 2 in Supplement 1). We examined B-MOUD initiations, defined as the first observed B-MOUD prescription after an OUD diagnosis, starting in January 2017.^5^ We coded facilities as having SCOUTT exposure if at least 1 PC-MH-P clinical team in the facility engaged in SCOUTT and all other facilities as non-SCOUTT. Time was measured by calendar year of B-MOUD dispensation.

We report the proportion of B-MOUD prescriptions and initiations dispensed by setting. Linear regressions, weighted by annual cohort size, were used to model differences in percentage of B-MOUD from PC-MH-P vs SUD within SCOUTT and non-SCOUTT facilities using group-time interaction. R, version 4.4.1 was used for analyses. Two-sided *P* < .05 was considered significant.

## Results

**Figure 1.**
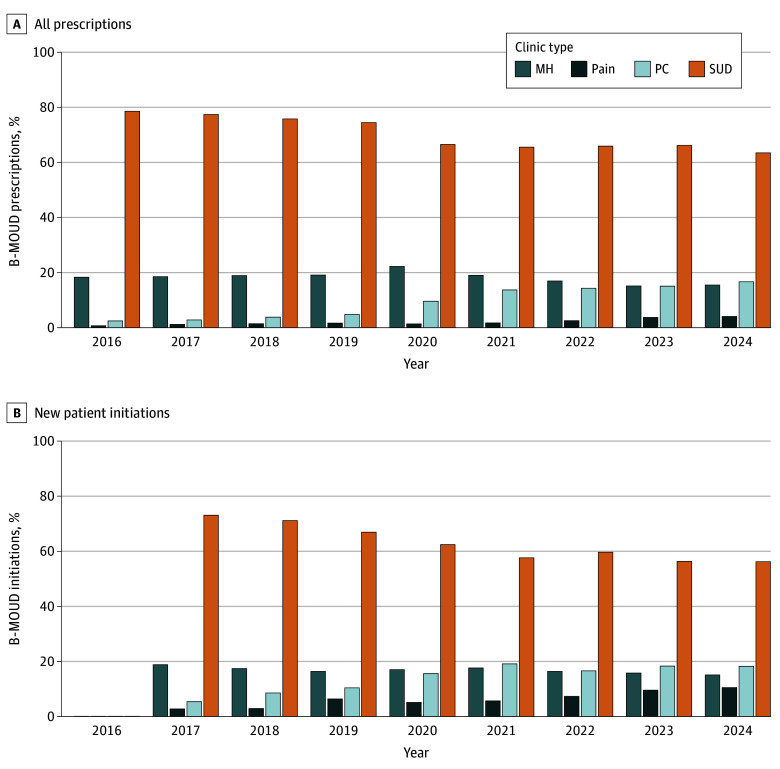
Settings of Buprenorphine Medication for Opioid Use Disorder (B-MOUD) Prescriptions (2016-2024) and Initiations (2017-2024) Prescriptions were counted in the year they were dispensed. Individuals may have contributed multiple prescriptions within the same year for all prescriptions and only once for initiations. MH indicates mental health; PC, primary care; and SUD, substance use disorder.

We identified 1 786 533 B-MOUD prescriptions for 46 267 veterans, 28 921 of which were initiations, in 56 SCOUTT and 918 non-SCOUTT facilities. Between 2016 and 2024, SUD settings provided the most B-MOUD prescriptions annually (Figure 1); however, their share decreased from 78.5% to 63.5% (*P* < .001). Similarly, the percentage of B-MOUD initiations from SUD settings decreased from 73.1% to 56.2% (*P* < .001).

**Figure 2.**
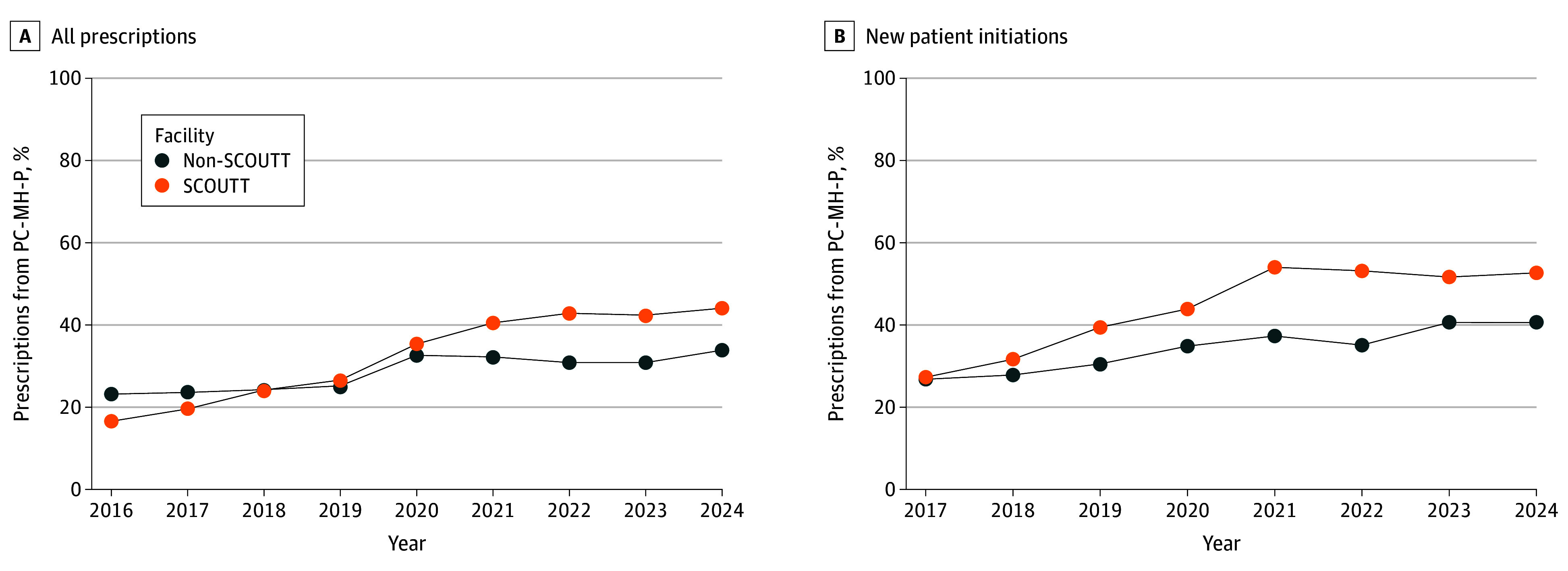
Prescribing and Initiating of Buprenorphine Medication for Opioid Use Disorder in Primary Care, Mental Health, and Pain (PC-MH-P) Clinics vs Substance Use Disorder Clinics in Stepped Care for Opioid Use Disorder Train the Trainer (SCOUTT) Initiative and Non-SCOUTT Facilities

In SCOUTT facilities, the percentage of B-MOUD prescriptions from PC-MH-P settings was 16.6% in 2016 and 44.1% in 2024 (Figure 2). In non-SCOUTT facilities, these percentages were 23.2% in 2016 and 33.9% in 2024. The mean yearly percentage-point change was greater in SCOUTT facilities than non-SCOUTT facilities (3.8 vs 1.4; *P* < .001). Similar trends were observed for B-MOUD initiations (3.9 vs 2.1; *P* = .03).

## Discussion

In this study, provision of B-MOUD rapidly shifted toward PC-MH-P settings from SUD settings and greater shifts were observed in VA facilities participating in SCOUTT. A train-the-trainer intervention, such that trained prescribers are equipped to spread B-MOUD knowledge and prescribing practices in their health system, may have played a role in accelerating the integration of B-MOUD prescribing into more accessible health care settings. Limitations are that prescribing from non-VA facilities was not examined, electronic health records may be subject to misclassification, and selection bias cannot be ruled out. Policies and initiatives launched in the VA may be a model to encourage B-MOUD prescribing in PC-MH-P settings in other large health care systems.

## Notes

Supplement 1. **eTable 1.** Included OUD Diagnosis *ICD-10* Codes **eTable 2.** Setting Categorization by 3-Digit Location Code Identifiers

Supplement 2.
Data Sharing Statement
