# Supplementary material for: Buprenorphine Treatment for Opioid Use Disorder in Non–Addiction Specialty Settings
Source: JAMA Netw Open. 2025 Nov 13;8(11):e2543543. doi: 10.1001/jamanetworkopen.2025.43543 (PMC12616456; doi:10.1001/jamanetworkopen.2025.43543)
Supplement: Supplement 1. — eTable 1. Included OUD Diagnosis ICD-10 Codes eTable 2. Setting Categorization by 3-Digit Location Code Identifiers [file jamanetwopen-e2543543-s001.pdf]

Supplemental Online Content

Huebler S, Jones AL, Zhao H, et al. Buprenorphine treatment for opioid use disorder in non–addiction specialty settings. *JAMA Netw Open.* 2025;8(11):e2543543. doi:10.1001/jamanetworkopen.2025.43543

eTable 1. Included OUD Diagnosis *ICD-10* Codes

eTable 2. Setting Categorization by 3-Digit Location Code Identifiers

This supplemental material has been provided by the authors to give readers additional information about their work.

© 2025 Huebler S et al. *JAMA Network Open*.

eTable 1. Included OUD Diagnosis *ICD-10* Codes

ICD-10 Code	Description
Opioid abuse/dependence	
F11.10	Opioid abuse, uncomplicated
F11.120	Opioid abuse with intoxication, uncomplicated
F11.121	Opioid abuse with intoxication, delirium
F11.122	Opioid abuse with intoxication, with perceptual disturbance
F11.129	Opioid abuse with intoxication, unspecified
F11.14	Opioid abuse with opioid-induced mood disorder
F11.150	Opioid abuse with opioid-induced psychotic disorder, with delusions
F11.151	Opioid abuse with opioid-induced psychotic disorder, with hallucinations
F11.159	Opioid abuse with opioid-induced psychotic disorder, unspecified
F11.181	Opioid abuse with opioid-induced sexual dysfunction
F11.182	Opioid abuse with opioid-induced sleep disorder
F11.188	Opioid abuse with other opioid-induced disorder
F11.19	Opioid abuse with unspecified opioid-induced disorder
F11.20	Opioid dependence, uncomplicated
F11.21	Opioid dependence, in remission
F11.220	Opioid dependence with intoxication, uncomplicated
F11.221	Opioid dependence with intoxication, delirium
F11.222	Opioid dependence with intoxication, with perceptual disturbance
F11.229	Opioid dependence with intoxication, unspecified
F11.23	Opioid dependence with withdrawal
F11.24	Opioid dependence with opioid-induced mood disorder
F11.250	Opioid dependence with opioid-induced psychotic disorder, with delusions
F11.251	Opioid dependence with opioid-induced psychotic disorder, with hallucinations
F11.259	Opioid dependence with opioid-induced psychotic disorder, unspecified
F11.281	Opioid dependence with opioid-induced sexual dysfunction
F11.282	Opioid dependence with opioid-induced sleep disorder
F11.288	Opioid dependence with other opioid-induced disorder
F11.29	Opioid dependence with unspecified opioid-induced disorder

© 2025 Huebler S et al. *JAMA Network Open*.

eTable 2. Setting Categorization by 3-Digit Location Code Identifiers

Type of care	Stop code/Stop code combinations
Primary care	• Primary stop code in (170, 171, 172, 173, 174, 175, 176, 177, 178, 156, 157, 322, 323, 338, 350, 348, 534, or 539) while secondary stop code NOT in (107, 115, 152, 311, 333, 334, 999, 474, 430, 328, 321, 329, 435, 103, 147, 148, 169, 181, 182, 199, 199, 216, 221, 224, 229, 324, 325, 424, 425, 428, 444, 445, 446, 447, 448, 449, 450, 527, 528, 530, 536, 537, 542, 545, 546, 579, 584, 597, 611, 686)<br>• Primary stop code NOT in (107, 115, 152, 311, 333, 334, 999, 474, 430, 328, 321, 329, 435, 103, 147, 148, 169, 181, 182, 199, 199, 216, 221, 224, 229, 324, 325, 424, 425, 428, 444, 445, 446, 447, 448, 449, 450, 527, 528, 530, 536, 537, 542, 545, 546, 579, 584, 597, 611, or 686) while secondary stop code in (179, 322, 323, 350, 531, or 534)<br>• Primary stop code 224 AND secondary stop code 323<br>• Primary stop code 326 AND secondary stop code 350<br>• Primary stop code 527 AND secondary stop code 534
Mental health	• Primary stop code in (84, 502, 505, 506, 509, 510, 512, 516, 520, 521, 524, 525, 526, 527, 531-542, 546, 550-554, 557-559, 561-584, 589)
Pain	• Primary stop code 420
SUD/Addiction	• Primary stop code in (513, 514, 517, 518, 519, 523, 543, 544, 545, 547, 548, 560, 706, 721, 722, 723, 724)

© 2025 Huebler S et al. *JAMA Network Open*.
