# Supplementary material for: Buprenorphine Treatment for Opioid Use Disorder in Non–Addiction Specialty Settings
Source: JAMA Netw Open. 2025 Nov 13;8(11):e2543543. doi: 10.1001/jamanetworkopen.2025.43543 (PMC12616456; doi:10.1001/jamanetworkopen.2025.43543)
Supplement: Supplement 2. — Data Sharing Statement [file jamanetwopen-e2543543-s002.pdf]

Data Sharing Statement

Huebler. Buprenorphine Treatment for Opioid Use Disorder in Non–Addiction Specialty Settings. *JAMA Netw Open*. Published November 13, 2025.
doi:10.1001/jamanetworkopen.2025.43543

Data
Data available: No

Additional Information
Explanation for why data not available: Access to a limited, de-identified dataset can be made available pending ethical approval and in accordance with VA guidelines. Those wishing to access the study data may contact the corresponding author to discuss the VA data access approval process.
